# A Comparison of Hyper-Reflective Retinal Spot Counts in Optical Coherence Tomography Images from Glaucomatous and Healthy Eyes

**DOI:** 10.3390/jcm10204668

**Published:** 2021-10-12

**Authors:** Luciano Quaranta, Carlo Bruttini, Giovanni De Angelis, Silvia Montescani, Alberto Ardizzone, Andreas Katsanos, Carmela Carnevale, Francesco Oddone, Ivano Riva

**Affiliations:** 1 Department of Surgical & Clinical, Diagnostic and Pediatric Sciences, Section of Ophthalmology, University of Pavia-IRCCS Fondazione Policlinico San Matteo, 27100 Pavia, Italy; carlo.bruttini@hotmail.it (C.B.); gianni.deangelis@iol.it (G.D.A.); silvia.montescani@gmail.com (S.M.); albertardizz@gmail.com (A.A.); 2 Department of Ophthalmology, University of Ioannina, 451 10 Ioannina, Greece; andreakatbp@hotmail.com; 3 IRCCS-Fondazione Bietti, 00198 Rome, Italy; oculistacarmelacarnevale@gmail.com (C.C.); oddonef@gmail.com (F.O.); 4 Istituto Clinico Sant’Anna, 25127 Brescia, Italy; ivano.riva@virgilio.it

**Keywords:** glaucoma, hyper-reflective retinal spots, optical coherence tomography, visual field

## Abstract

Purpose: To compare the number of hyper-reflective retinal spots (HRS) in optical coherence tomography (OCT) images of healthy controls and patients affected with primary open angle glaucoma (POAG). Methods: Thirty patients affected with POAG and 34 healthy controls were recruited and underwent raster OCT examination of the macular region. Among the acquired B-scans, the one with the lowest foveal thickness was selected, and a central area of 3000 μm was defined (region of interest, ROI), in order to identify HRS. HRS were defined as small point-like hyper-reflective elements, detectable at the visual inspection of the OCT image. HRS were independently counted by two investigators in the ROI of each OCT scan. Results: Inter-rater agreement for HRS counting was good to excellent (ICC = 0.96, 95% CI: 0.83–0.99). More HRS were found in the OCT images from glaucoma patients, in comparison with healthy controls (average value: 90.5 ± 13.02 and 74.72 ± 11.35, for glaucoma and healthy subjects, respectively; *p* < 0.01). Significant correlations between the average number of HRS and visual field mean deviation (MD, *p* = 0.01) and pattern standard deviation (PSD, *p* < 0.01) were found. Conclusions: OCT images from glaucoma patients showed a higher number of HRS when compared with healthy controls. As HRS have been hypothesized to be a sign of neuroinflammation, these results may support the role of neuroinflammation in glaucoma etiopathogenesis.

## 1. Introduction

Glaucoma is a neurodegenerative disease consisting of a non-uniform group of ocular disorders characterized by a number of clinical features including visual field (VF) defects, retinal ganglion cell (RGC) death and progressive degeneration of the optic nerve. This optic neuropathy represents a complex, multifactorial disease, in which several molecular pathways are involved. Growing evidence suggests that the interaction of RGCs and glial cells, including microglial cells, retinal astrocytes and Muller cells, is critically important for the process of glaucomatous neurodegeneration [1,2]. Indeed, by exerting both neuro-supportive and detrimental effects, glial cells play a key role in determining the RGC life or death [3].

The environment created by various stress stimuli in the glaucomatous tissue is a major initiator of neuroinflammatory pathways [1]. In this regard, intraocular pressure (IOP) elevation has been described as one of the most important factors responsible for secondary reactions that activate microglia [4,5]. The plasticity of these cells makes them react to any homeostatic imbalance, by changing morphology and gene expression. In an initial, acute phase, microglial cells attempt to isolate and resolve the early neuronal damage, by incorporating degenerated material and exerting their phagocytic action against cell and axon debris [1]. However, chronically activated microglial cells stimulate several other neuroinflammatory cascades, which, in turn, are associated with secondary signal transduction pathways. These pathways increase the expression of pro-inflammatory cytokines and inflammatory mediators, including interleukin-1α, interleukin-6 and TNF-α, that are the hallmark of glaucoma neuroinflammation, ultimately leading to RGC degeneration [2,6].

The presence of activated retinal microglial cells has recently been associated with the presence of hyper-reflective spots (HRS) in macular B-scan images obtained by the means of optical coherence tomography (OCT) [7,8]. More HRS have been demonstrated in patients affected with ocular diseases involving the activation of neuroinflammatory cascades, such as age-related macular degeneration (AMD), diabetic retinopathy (DR) and retinal vein occlusion [7,9,10,11]. The clinical relevance of HRS is actually unknown. However, HRS may be considered a sign of neuroinflammation of the retina. Neuroinflammation has a relevant role in the etiopathogenesis of several ophthalmological diseases, and for this reason, HRS might be used as an OCT biomarker indicating of the state of disease activity, and of the efficacy of therapeutic treatments. Interestingly, Coscas et al., evaluating HRS in patients affected by AMD, found that the increase of HRS was the first relevant sign of disease reactivation at OCT [9]. The use of HRS as a disease biomarker would be of particular interest in glaucoma, in which anatomical damage generally precedes functional loss determined by VF examination. Such a biomarker could also be useful in determining the efficacy of glaucoma treatments, reducing neuroinflammation and rate of progression of the disease.

To the best of our knowledge, the presence and number of HRS in primary open angle glaucoma (POAG) patients have not been investigated. Aim of this study is to compare the number of HRS in the OCT macular B-scans of patients affected by POAG and healthy controls.

## 2. Subjects and Methods

This observational study was carried out at the Ophthalmology Clinic of the IRCCS Policlinico San Matteo Foundation and Hospital, Pavia, Italy. All participants were treated in accordance with the ethical tenets on human research of the Declaration of Helsinki. The approval of the local Ethics Committee was also obtained (protocol no. 20190103536). All participants signed an informed consent form, both for their participation in the study and for publication of the results.

### 2.1. Sample Population

Subjects already diagnosed with POAG were consecutively enrolled among the patients attending the Glaucoma Service of the abovementioned clinic. POAG was defined as the presence of an ophthalmoscopically abnormal optic disc (diffuse or focal thinning of the neuro-retinal rim), an open angle at gonioscopy (Shaffer grade III or IV), and the presence of an abnormal VF consistent with glaucoma. Inclusion criteria were an age between 50 and 80 years, at least 3 reliable and non-progressing VF tests in the previous 2 years (24-2 Sita Standard Program, Humphrey Visual Field Analyzer (HFA), Carl Zeiss Meditec, Dublin, OH, USA), and an IOP between 10 and 18 mm Hg at enrollment visit (Goldmann applanation tonometry). VF progression was assessed by means of the Guided Progression Analysis (GPA) software package of the HFA. GPA has been validated in previous studies, and is a simple and practical method to evaluate functional progression in patients affected with POAG [12,13,14]. Patients flagged by the GPA software as “possible progression” or “likely progression” were excluded from the study.

Allowed hypotensive topical medications were β-blockers, α2-adrenergic agents and carbonic anidrase inhibitors, administered as single agents or as fixed combinations. Patients under treatment with a prostaglandin analogue were excluded, due to the known pro-inflammatory effects of prostaglandins [15].

Controls were recruited from the healthy attendees of the outpatient service, typically scheduled for routine ophthalmological visits. Inclusion criteria were an age between 50 and 80 years and no sign or history of ophthalmological diseases.

Exclusion criteria for both the groups of subjects affected with POAG and healthy controls were: a diagnosis of diabetes and/or autoimmune diseases, myopia higher than 3 diopters, media opacities preventing a reliable ophthalmological and OCT examination, cataract surgery performed within the previous 12 months, lifetime history of any other intraocular surgery.

### 2.2. Interventions

All participants underwent a complete ophthalmological examination, including best-corrected visual acuity, biomicroscopy of the anterior segment, IOP measurement with Goldmann applanation tonometry, fundus evaluation after pupil dilation with 1% tropicamide eye drops, and OCT analysis of the macular region with Spectralis OCT (Heidelberg Engineering GmbH, Heidelberg, Baden-Württemberg, Germany). All OCT scans were acquired using the Automatic Real-Time mode of Spectralis OCT, adjusted to 20 frames. To be eligible for the analysis, OCT scans had to show a quality score above 25 dB.

**Figure 1:**
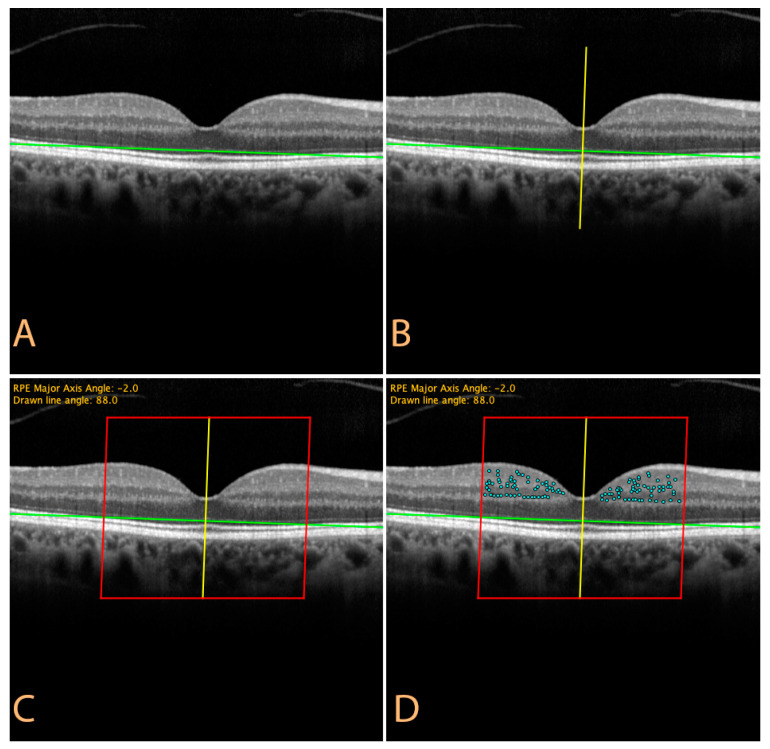
OCT image processing by ImageJ script (available as Supplementary Materials); (**A**) Identification of the retinal pigment epithelium major axis; (**B**) A line passing through the center of the fovea is drawn (reference line), according to the angle suggested by the script: (**C**) Two lines are automatically superimposed, 1500 µm apart of the reference line, temporally and nasally, defining a region of interest (ROI); (**D**) Hyper-reflective retinal spots are counted by the investigator inside the newly defined ROI.

Spectralis OCT is a spectral domain OCT incorporating a confocal scanning laser ophthalmoscope, capable of acquiring posterior pole B-scans with a resolution of approximately 7 µm. For study purposes, a standard volume analysis comprising of 25 equally spaced horizontal B-scans was acquired. Among the acquired B-scans, the one with the thinnest foveal thickness, as determined by the Spectralis OCT software, was selected and imported into the ImageJ software as a “.tiff image” [16]. A custom Python script for ImageJ (Supplementary Materials) was written, in order to process the image before the analysis. The script asks the user to mark the nasal and temporal retinal pigment epithelium (RPE) margins, in order to identify the RPE major axis and extrapolate its angle (Figure 1A). The user is then asked to draw a line passing though the center of the fovea, according to the prompted angle value (i.e., perpendicular to the RPE major axis, Figure 1B). Two parallel lines are then automatically drawn 1500 um apart of the reference line, temporally and nasally, defining a region of interest (ROI, Figure 1C, red rectangle).

### 2.3. HRS Counting

HRS were counted in the ROI of each processed image, after 2× image magnification, by two independent, masked investigators. HRS were defined as isolated, punctiform (≤30 µm), and moderately reflective (i.e., similar to the RNFL) spots with no back shadowing [10]. They were counted in all the retinal layers, with the exception of the outer nuclear layer, the EPR and the choroid.

### 2.4. Statistical Analysis

Unit of analysis was the eye. If both eyes were eligible, one eye was randomly chosen for the analysis. Continuous variables were described as mean and standard deviation (SD), and were tested for normality using a Shapiro–Wilk test. Categorical variables were reported as frequency and percentage. A Mann–Whitney-Wilcoxon rank sum test was used to compare population characteristics at enrollment.

The intra- and inter-rater agreement was evaluated by means of the intraclass correlation coefficient (ICC). OCT scans acquired from the first 10 enrolled subjects were used for inter-rater agreement evaluation. For intra-rater agreement assessment, OCT images from the first 5 enrolled subjects were used. In this last case, each investigator analyzed the same image 5 times, for a total of 50 measurements (25 measurements per investigator). A two-way ICC model was used for all the analyses. An ICC value less than 0.5 is indicative of poor reliability, values between 0.5 and 0.75 indicate moderate reliability, values between 0.75 and 0.9 indicate good reliability, and values greater than 0.90 indicate excellent reliability [17].

An independent t-test or a Mann–Whitney–Wilcoxon rank sum test was used to compare HRS numerosity in the glaucoma and control group, according to data distribution. Comparisons were performed for each investigator and for the average counts of the two investigators. A sample size of 28 subjects per group was calculated, in order to achieve a 80% power to detect a difference between groups of 10 HRS, with a pooled standard deviation of 13 and an α-error of 0.05.

IOP, age and VF mean deviation (MD) and pattern standard deviation (PSD) were correlated to the average HRS numerosity (i.e., the average count of the two investigators) in the entire study group. In this analysis, a Pearson correlation test was used for normally distributed data, otherwise a Spearman’s rank correlation test was performed.

Significance level was set at 0.05. All analyses were performed using an in-house software library based on the Python SciPy environment [18] and with JMP statistical software, Version 14, SAS Institute Inc., Cary, NC, USA, 1989–2021.

## 3. Results

Thirty-four eyes of 34 healthy controls and 30 eyes of 30 POAG patients were enrolled (Table 1). Mean age was 64.18 ± 10.40 and 67.43 ± 13.07, for the control and the POAG groups, respectively (*p* = 0.08). All enrolled subjects were Caucasian. Mean IOP for controls and glaucoma patients was 14.56 ± 2.16 mm Hg and 15.30 ± 5.02 mm Hg, respectively (*p* = 1.00).

Inter-rater agreement for HRS counting was good to excellent (ICC = 0.96, 95% CI: 0.83–0.99), while intra-rater agreement was good to excellent for the first investigator (ICC: 0.96, 95% CI: 0.88–0.99) and moderate to excellent for the second investigator (ICC: 0.86, 95% CI: 0.62–0.98). HRS were detected in 100% of the eyes. A greater number of HRS were detected in the OCT images from POAG eyes, when compared with healthy controls (*p* < 0.01, Table 2). These results were confirmed for each investigator and for the average counts of the two investigators.

**Figure 2:**
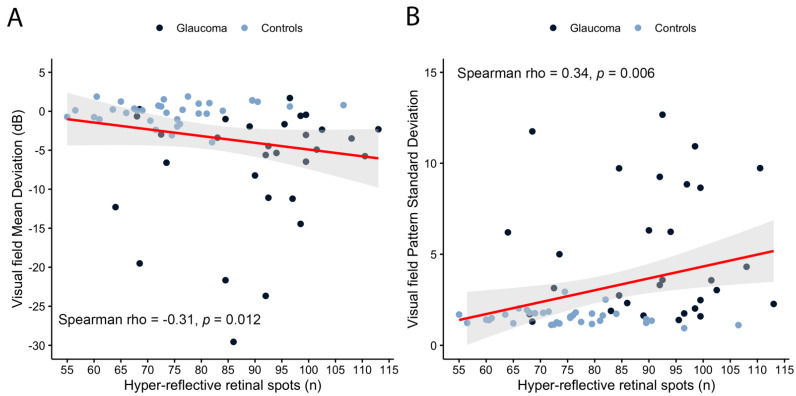
Scatterplot of hyper-reflective retinal spot number vs. visual field mean deviation (MD, (**A**)) and pattern standard deviation (PSD, (**B**)). Spearman’s rank correlation test.

Significant correlations between HRS number and MD (Spearman’s rho: −0.31, *p* = 0.01, Figure 2A) and between HRS number and PSD (Spearman’s rho: 0.34, *p* < 0.01, Figure 2B) were found. Instead, age (Pearson r: 0.16, *p* = 0.19) and IOP (Spearman’s rho: −0.04, *p* = 0.71) were not correlated to HRS numerosity.

## 4. Discussion

In the present study, a significant greater number of HRS was found in the OCT images from POAG eyes, when compared with healthy controls. Considering that HRS have been referred as a marker of microglial activation [7,8], our data are consistent with experimental evidence regarding the role of neuro-inflammation and neurodegeneration in glaucoma [2].

HRS are a relatively new OCT finding. An increased number of HRS was first described in OCT scans from patients affected with AMD by Coscas et al., who suggested HRS may represent activated microglial cells [19]. In a further study, the same group of authors found a reduction of the HRS numerosity in OCT scans from AMD patients treated with intravitreal injections of anti-vascular endothelial growth factor (anti-VEGF), and identified the increase of HRS as the first relevant sign of disease re-activation at OCT [9]. Vujosevic et al. analyzed a set of OCT scans from healthy controls and diabetic patients with and without mild DR, i.e., without macular edema and intraretinal exudation [8]. Patients affected with DR had higher HRS counts than diabetic patients without ophthalmological signs of the disease, and these in turn had more HRS than healthy controls. As a result, the number of HRS progressively increased according to the clinical evolution of the disease, potentially being an expression of its pathogenesis and of neuroinflammation. HRS have also been described in other patients with diabetic macular edema and retinal vein occlusion [7,20]. In these cases, however, different hypotheses have been offered to explain their presence, such as the fact that HRS may represent lipoprotein extravasation as a precursor of hard exudates in diabetic macular edema [20], or sub-clinical leakage of blood constituents in retinal vein occlusion [7]. Finally, HRS have been recently investigated as a potential sign of retinal neuroinflammation after uncomplicated cataract surgery by Pilotto et al. [21].

Although the exact nature of HRS remains unknown, the hypothesis that they may represent, at least in some eyes, activated and aggregated microglial cells is consistent. HRS have been described, both in the early stages of DR [8] and immediately after cataract surgery [21], at the level of the inner retinal layers, where resting microglia is normally resident, and where the inflammation process begins. As the disease progresses, in the case of DR, or the time passes, after cataract surgery, HRS can also be found inside the outer retinal layers, suggesting their migration through the retinal architecture [8,21]. This behavior mimics the migration of microglial cells from the inner to the outer retina, as previously described in histopathological studies on neuroinflammation [22,23]. In case of retinal injury, microglial activation causes an increase of the adhesiveness of Müller cells, that act as an adhesive scaffold for microglia migration across the retina [23]. Interestingly, in naïve patients affected with DR and macular edema, Ceravolo et al. recently demonstrated that an intravitreal implant of dexamethasone 0.7 mg is more effective than monthly intravitreal injections of ranibizumab 0.5 mg, at reducing the number of HRS in swept source OCT images (*p* = 0.01) [24]. These results further support the concept that HRS may represent an indirect sign of neuroinflammation, being more impacted by corticosteroid than anti-VEGF treatment.

Several histopathological and animal model studies have suggested a link between neuroinflammation and glaucoma, [2] being glial cells, and especially microglia, a key factor in this respect [1,2,3]. It has been demonstrated that glaucomatous injury and IOP elevation are capable of triggering microglia activation [23]. Activated microglia undergo complex interactions with macroglia, comprising astrocytes and Müller cells, ultimately bringing to cellular phenotype switch and intra-retinal migration [23]. Activated microglia shift to an ameboid cellular morphology and a predominantly pro-inflammatory phenotype, releasing cytokines and chemokines, such as TNF-α, IL-6, and complement components [25]. According with these data, increased levels of inflammatory mediators have been demonstrated inside the retina and in the aqueous humor of glaucomatous patients and in experimental models of glaucoma [26]. This environment is thought to start and sustain the RGC degeneration process.

The finding of an increased number of HRS in the central retina of POAG patients, when compared to controls, supports the hypothesis that HRS may represent, in these eyes, foci of activated microglia. HRS were counted at the level of the inner retina, i.e., between the internal limiting membrane and the outer plexiform layer, because these retinal strata, comprising the ganglionar cell layer and the retinal nerve fiber layer, are the most affected in the glaucomatous disease [27]. IOP injury is primarily exerted at this level [28], and RGC degeneration is the hallmark of the glaucomatous pathogenetic process. Moreover, the inner retinal layers constitute the site of the initial microglia activation [23], where HRS may also be easily found in the early stages of the disease. Notably, all glaucoma patients enrolled in this study were on treatment with topical hypotensive drugs. Although topical prostaglandin analogues were an exclusion criterium for enrollment, due to their well-known pro-inflammatory characteristics [15], we cannot exclude a direct effect of other hypotensive drugs or preservatives on the retina. Indeed, previous studies have found a potential pro-inflammatory effect on the retina of topically applied benzalkonium chloride, a preservative commonly used in eye-drops [29]. Moreover, α2-adrenergic agents are known to exert a neuroprotective effect on RGCs in vitro [30,31], in animal models of glaucoma [32,33] and potentially in human subjects affected with normal tension glaucoma [34]. However, the aim of this pilot study was uniquely to investigate the numerosity of HRS in patients affected with glaucoma in comparison to healthy controls, and is underpowered to find differences among patients treated with different topical hypotensive agents. On the other hand, the finding of a significant difference of HRS numerosity between glaucoma patients and controls, in spite of a potential neuroprotective agent, theoretically reducing neuroinflammation, administered to about the 48% of enrolled POAG patients, confirm and strengthen the results of this study.

Significant correlations were found between the number of HRS and the MD and PSD value. More specifically, the HRS number increased with the decrease of the MD value, and with the increase of the PSD value. According to these results, more damaged VF tests were associated with a higher number of HRS. This is an interesting finding, suggesting a possible correlation between structure and function, with functional damage increasing along with retinal neuroinflammation. Although this hypothesis is intriguing, dispersion of data in our analysis does not allow us to draw definitive conclusions on the topic, and studies with bigger sample size are required. In the case where these results would be confirmed, HRS may represent an in vivo biomarker of the glaucomatous disease, disclosing several possibilities, including an early pre-functional diagnosis, the evaluation of the state of the disease, and the evaluation of its response to different treatments.

In the present study, the number of HRS was generally higher than previously reported, both in healthy controls [10] and in patients affected by other eye diseases [7,8,9]. This discrepancy may be due to several factors. First, various definitions of HRS have been used in different studies [7,9,10,20], obviously affecting the identification of HRS and their counting. Second, the area in which HRS should be searched has not been uniquely defined, varying from study to study [9,10,21]. In the present investigation, a script was developed to define a ROI, equally spaced around the fovea and comprising 3000 μm of central retina, where HRS were counted. This area is generally wider than the ones used in the previous studies [8,10]. The script is attached as Supplementary Materials, and can be easily used by other authors in further studies. Third, other methodological differences, such as searching for HRS prevalently next to pathological lesions [7,9,20], or with/without retinal image magnification [10], may be sources of additional counting variability.

The study has certain limitations. Indeed, the ability of different OCT instruments to detect HRS may vary, and the present results might not apply to all commercially available OCT platforms. Moreover, even if counting HRS within a given OCT scan is straightforward, rater variability may obviously affect the results. Future technical developments in OCT imaging may allow for the automatic determination of HRS counts.

## 5. Conclusions

HRS evaluation in macular OCT B-scans seems to be a promising field of research in glaucoma, and could lead to a novel OCT biomarker of inflammatory processes occurring in glaucoma patients. Further studies are needed, with larger sample sizes, in order to confirm present results and explore their potential clinical implications.

## Figures and Tables

**Table 1:** Study population characteristics.

Variable	Controls (*n* = 34)	Glaucoma (*n* = 30)	*p*-Value ^#^
Age (SD)	64.18 (10.40)	67.43 (13.07)	0.08
IOP (SD)	14.56 (2.16)	15.30 (5.02)	1.00
Visual field MD (dB)	−0.07 (1.38)	−7.09 (7.74)	<0.01
Visual field PSD	1.56 (0.41)	4.98 (3.55)	<0.01
Topical treatment			
β-blockers–*n* (%)	-	24 (77.4)	
α2-adrenergic agents–*n* (%)	-	15 (48.38)	
Carbonic anhydrase inh.–*n* (%)	-	14 (45.16)	

SD: Standard deviation; MD: Mean deviation; PSD: Pattern standard deviation; ^#^: Mann–Whitney–Wilcoxon rank sum test.

**Table 2:** HRS counts according to investigator 1 and investigator 2.

	Glaucoma	Controls	*p*-Value
Investigator 1, *n* (SD)	90.20 (15.06)	75.79 (14.14)	<0.01 *
Investigator 2, *n* (SD)	90.80 (13.80)	73.65 (10.83)	<0.01 ^#^
Average, *n* (SD)	90.50 (13.02)	74.72 (11.35)	<0.01 *

SD: Standard deviation; *: Two sample independent t-test; ^#^: Mann–Whitney–Wilcoxon rank sum test.

## Data Availability

The data presented in this study are available on request from the corresponding author. The data are not publicly available due to team decision.

## Supplementary Materials

The following are available online at https://www.mdpi.com/article/10.3390/jcm10204668/s1. OCT HRS Python script for ImageJ—A python script for ImageJ Fiji for Mac (version 2.0/1.53c) to define a standardized region of interest in the OCT macular B-scan image, measuring 3000 μm and centered on the fovea.
