# Supplementary material for: A Comparison of Hyper-Reflective Retinal Spot Counts in Optical Coherence Tomography Images from Glaucomatous and Healthy Eyes
Source: J Clin Med. 2021 Oct 12;10(20):4668. doi: 10.3390/jcm10204668 (PMC8538917; doi:10.3390/jcm10204668)
Supplement: Supplementary file 1 [file jcm-10-04668-s001.zip › jcm-1352039-supplementary.pdf]

```
#This python script asks the user to mark the nasal and
#temporal margins of the RPE in the macular B-scan OCT image.
#It automatically draws the RPE major axis and extrapolates its angle.
#The user is then asked to draw a line perpendicular to the RPE major
#axis, according to the prompted angle. The line has to pass through
#the center of the fovea (reference line, RL). The script then defines
#a region of interest (ROI), comprising the retina 1500 um on the left
#and on the right of the RL.
#Script tested with Spectralis OCT (Heidelberg Engineering GmbH,
#Heidelberg, Baden-W√°rttemberg, Germany)

#Written by Ivano Riva, MD.

from ij.gui import Roi, Overlay, Line, GenericDialog
from ij.plugin.frame import RoiManager
from ij.measure import ResultsTable
from ij import IJ, ImagePlus, WindowManager
from ij.gui import WaitForUserDialog
from ij.gui import TextRoi
from java.awt import Font

ov = Overlay()

#Get scale
imp = IJ.getImage()
imp.setRoi(500, 430, 75, 66)
IJ.run("Duplicate...", "title=Scale_set.tif")
IJ.run("Set... ", "zoom=1000")
IJ.run("Set Scale...", "distance=0 known=0 unit=pixel")
IJ.run("Point Tool...", "type=Hybrid color=Green size=Large label
counter=0")
IJ.setTool("line");
WaitForUserDialog("Please draw a line with an angle of zero along the
horizontal arm of the scaler.\nClick ok when done.").show()

imp2 = IJ.getImage()
scale = imp2.getRoi()
imp2.setRoi(scale)
scale_length = scale.getLength()

shift = (1500 * scale_length)/200

IJ.selectWindow("Scale_set.tif");
IJ.run("Close");
IJ.run("Select None");

#Crop the image
imp = IJ.getImage()
IJ.run("Set Scale...", "distance=0 known=0 unit=pixel")
imp.setRoi(630, 15, 470, 460)
IJ.run("Crop");

#Get the RPE major axis and extrapolate its angle
Window_title = imp.getTitle()
IJ.run("Point Tool...", "type=Hybrid color=Green size=Large label
counter=0");
IJ.setTool("multipoint")
WaitForUserDialog("Please mark the nasal and temporal margins of the
RPE.\nWhen done, click OK.").show();
```

```
IJ.run(imp, "Measure", "");
table = ResultsTable.getResultsTable()
y1 = table.getValue("Y", 0)
y2 = table.getValue("Y", 1)
x1 = table.getValue("X", 0)
x2 = table.getValue("X", 1)

line = Line(x1,y1, x2, y2)
line.setStrokeWidth(2)
imp.setRoi(line)
ov.add(line)
imp.setOverlay(ov)
IJ.run("Flatten")

Angle_line = round(line.getAngle(line.x1, line.y1, line.x2, line.y2), 1)

if Angle_line <= 0:
      Angle_draw = -(90 + abs(Angle_line))
else:
      Angle_draw = -(90 - abs(Angle_line))

Angle_draw_str = str(Angle_draw)
Angle_line_str = str(Angle_line)
IJ.selectWindow("Results");
IJ.run("Close");
IJ.selectWindow(Window_title);
IJ.run("Close");

ov.clear()

#Ask the user to draw a reference line, according to the suggested angle
imp = IJ.getImage()
Window_title1 = imp.getTitle()
IJ.run("Point Tool...", "type=Hybrid color=Yellow size=Large label
counter=0");
IJ.setTool("line");

ls = "Please draw a line perpendicular to the RPE:\n \n- Start from the
inner foveal retinal border\n- Draw the line according to the suggested
angle\n- Move the line to center on the fovea\n- Click ok when done.\n
\nSuggested angle (perpendicular to RPE): " + Angle_draw_str
WaitForUserDialog(ls).show();
roi1 = imp.getRoi()
imp.setRoi(roi1)
roi1.setStrokeWidth(2)
ov.add(roi1)
imp.setOverlay(ov)
Angle2 = str(round((roi1.getAngle() + 180),1))

polygon = roi1.getFloatPoints()
x3 = polygon.xpoints[0]
x4 = polygon.xpoints[1]
y3 = polygon.ypoints[0]
y4 = polygon.ypoints[1]

#Draw the ROI
IJ.run("Flatten")
IJ.selectWindow(Window_title1);
IJ.run("Close");
ov.clear()
```

```
imp = IJ.getImage()
Window_title2 = imp.getTitle()
IJ.run("Point Tool...", "type=Hybrid color=Red size=Tiny label
counter=0");
line2 = Line((x3+shift), y3, (x4+shift),y4)
line3 = Line((x3-shift), y3, (x4-shift),y4)
line4 = Line((x3-shift), y3, (x3+shift), y3)
line5 = Line((x4-shift), y4, (x4+shift), y4)
line2.setStrokeWidth(2)
line3.setStrokeWidth(2)
line4.setStrokeWidth(2)
line5.setStrokeWidth(2)
imp.setRoi(line2)
imp.setRoi(line3)
imp.setRoi(line4)
imp.setRoi(line5)
IJ.run("Select None");
ov.add(line2)
ov.add(line3)
ov.add(line4)
ov.add(line5)
imp.setOverlay(ov)
IJ.run("Select None");
IJ.run("Flatten");
IJ.selectWindow(Window_title2);
IJ.run("Close");

ov.clear()
imp = IJ.getImage()
IJ.run("Point Tool...", "type=Hybrid color=Orange size=Large label
counter=0");
font = Font("SansSerif", Font.PLAIN, 12)
test1 = "RPE Major Axis Angle: " + Angle_line_str
test2 = "Drawn line angle: " + Angle2
roi = TextRoi(5, 5, test1, font)
roi1 = TextRoi(5, 20, test2, font)
imp.setRoi(roi)
imp.setRoi(roi1)
ov.add(roi)
ov.add(roi1)
imp.setOverlay(ov)
IJ.run("Select None")
```
